# Chemo-morphological evaluation of the blending behaviour of PMB and polymer-modified RAP binder

**DOI:** 10.1617/s11527-026-03201-6

**Published:** 2026-07-20

**Authors:** Isabeau Kokken, Bowen Li, Antonio Roberto, Johannes Mirwald, Wim Van den Bergh, Bernhard Hofko

**Affiliations:** 1 Sustainable Pavements and Asphalt Research (SuPAR), Faculty of Applied Engineering, University of Antwerp, 2020 Antwerp, Belgium; 2 Christian Doppler Laboratory for Chemo-Mechanical Analysis of Bituminous Materials, Institute of Transportation, TU Wien, 1040 Vienna, Austria; 3 EMPA, Ueberlandstrasse 129, Dübendorf, CH-8600 Switzerland

**Keywords:** Polymer-modified binder, Reclaimed asphalt pavement, Bitumen blending, SARA fractions, Fluorescence microscopy, Recycling

## Abstract

Polymer-modified reclaimed asphalt pavement (PM-RAP) is increasingly available for recycling, but the complex ageing and degradation of styrene-butadiene-styrene (SBS)-modified binders hinder its effective reuse in surface layers. This paper aims to understand the chemo-morphological blending behaviour between aged PM-RAP binders and fresh polymer-modified bitumen (PMB) at recycling ratios of 30% and 60%. Three PMBs from different geographical locations were combined with PM-RAP binders originating from the same respective regions. In total, three PMBs and three corresponding PM-RAP sources were used. Chemical characterisation was performed using Fourier-Transform Infrared (FTIR) spectroscopy, SARA fractionation, and FTIR analysis of the individual fractions. Morphological analysis was carried out using fluorescence microscopy. Results show that the addition of PM-RAP binder increases the ageing index and alters SBS-related signals depending on the RAP source. SARA analysis revealed a shift toward higher resin and asphaltene content with increasing PM-RAP content, while FTIR of the fractions indicated that a majority of the SBS remains in the n-heptane-insoluble phase, together with the asphaltenes. Blending occurred primarily through physical mixing, and strong agreement between experimental and calculated blend compositions (*R*^2^ > 0.9 relative to the *X* = *Y* parity line) suggests potential for predictive recycling. Fluorescence microscopy confirmed morphological changes reflected by a reduction in fluorescence intensity and polymer domain size upon increased PM-RAP binder percentages, highlighting limitations of chemical analysis alone. These findings provide new insights into the interaction mechanisms between aged and virgin SBS-modified binders and act as a foundation for more targeted recycling strategies.

## Introduction

The combined effects of rising traffic volumes and extreme climate conditions place increasing stress on pavements, causing problems such as rutting, cracking, and ravelling, which lead to increased maintenance and re-paving of surface asphalt layers. To enhance the performance of asphalt pavements under such conditions, polymers are often added to bitumen due to their elastic properties. Among these, styrene-butadiene-styrene (SBS) is one of the most widely used because it forms a crosslinked network within the binder, where the rigid polystyrene blocks add strength and the rubbery polybutadiene sections add elasticity [1, 2].

According to the European Asphalt Pavement Association (EAPA), around 18% of total bitumen production in the European Union is modified [3]. While this percentage has remained relatively stable in recent years, the cumulative length of roads paved with polymer-modified bitumen (PMB) is steadily increasing. As these pavements reach the end of their service life, growing volumes of polymer-modified reclaimed asphalt pavement (PM-RAP) are entering the recycling stream. However, the incorporation of RAP into surface layers is often restricted to low percentages (e.g. 20% for private works in Belgium [4]). This results in the downcycling of high-quality aggregates and aged PMB, both expensive components. At the mixture scale, only a limited number of studies have investigated the challenges of recycling PM-RAP, with the most common issues being decreased cracking resistance, fatigue resistance and moisture resistance upon RAP addition [5–9]. On the binder scale, a key challenge is the degradation of SBS polymers and the oxidation of the binder during the pavement’s service life. While polymer degradation and binder ageing have been studied extensively [10–13], their impact on the blending behaviour and compatibility between aged and virgin PMBs remains poorly understood. Making things more complicated, the condition of the polymer at the end of life depends on a range of factors, including polymer type and concentration, pavement service time, and environmental exposure. Consequently, no RAP source is the same, and even within a single RAP source, considerable inhomogeneity can exist [14, 15].

Studies by Liu et al. [16, 17] showed that while ageing reduces the effectiveness of SBS in reclaimed binders, traces of the polymer remain active and can still influence the performance of blends with virgin binders. However, restoring the original chemical characteristics remained a challenge. Margaritis et al. [18] remarked a dilution of the polymer-rich phase and minimal residual polymer activity at higher RAP contents, further highlighting the complexity of PMB recycling. Bocci et al. [19] reported that a 50% blend of a highly modified asphalt (HiMA) binder with PM-RAP exhibited good fatigue performance and resistance to permanent deformation, suggesting that the higher SBS content in HiMA can positively influence the rheological behaviour of the blend. It is important to note that blending studies at the binder scale assume full blending of the RAP binder with the PMB. However, mixture-scale studies have shown that full blending is usually not achieved [20–22].

It is known that the chemical and morphological composition of bitumen has a strong influence on its mechanical properties. In polymer-modified binders, the ratio of saturates, aromatics, resins, and asphaltenes (SARA) is important both for mechanical behaviour and for compatibility with the polymer [13, 23]. Fourier-Transform Infrared spectroscopy (FTIR) can be used to monitor changes in polarity, oxidation, and the presence of polymer-related functional groups [24, 25], but it is limited in that it cannot evaluate the intactness of the polymer network structure. Fluorescence microscopy helps visualise the dispersion of the polymer within the bitumen phase, which directly affects rheological performance [23, 26–28].

Understanding these aspects is essential before making any modifications to aged binders, such as adding rejuvenators, softer binders, or extra polymer. Despite these insights, a detailed chemo-morphological understanding of how aged PMB from RAP interacts with virgin PMB during blending is still lacking. To address this gap, this study investigates the interaction and compatibility between aged and virgin PMBs across different blend ratios using combined spectroscopic and microscopic techniques.

## Objectives and scopes

**Fig. 1:**
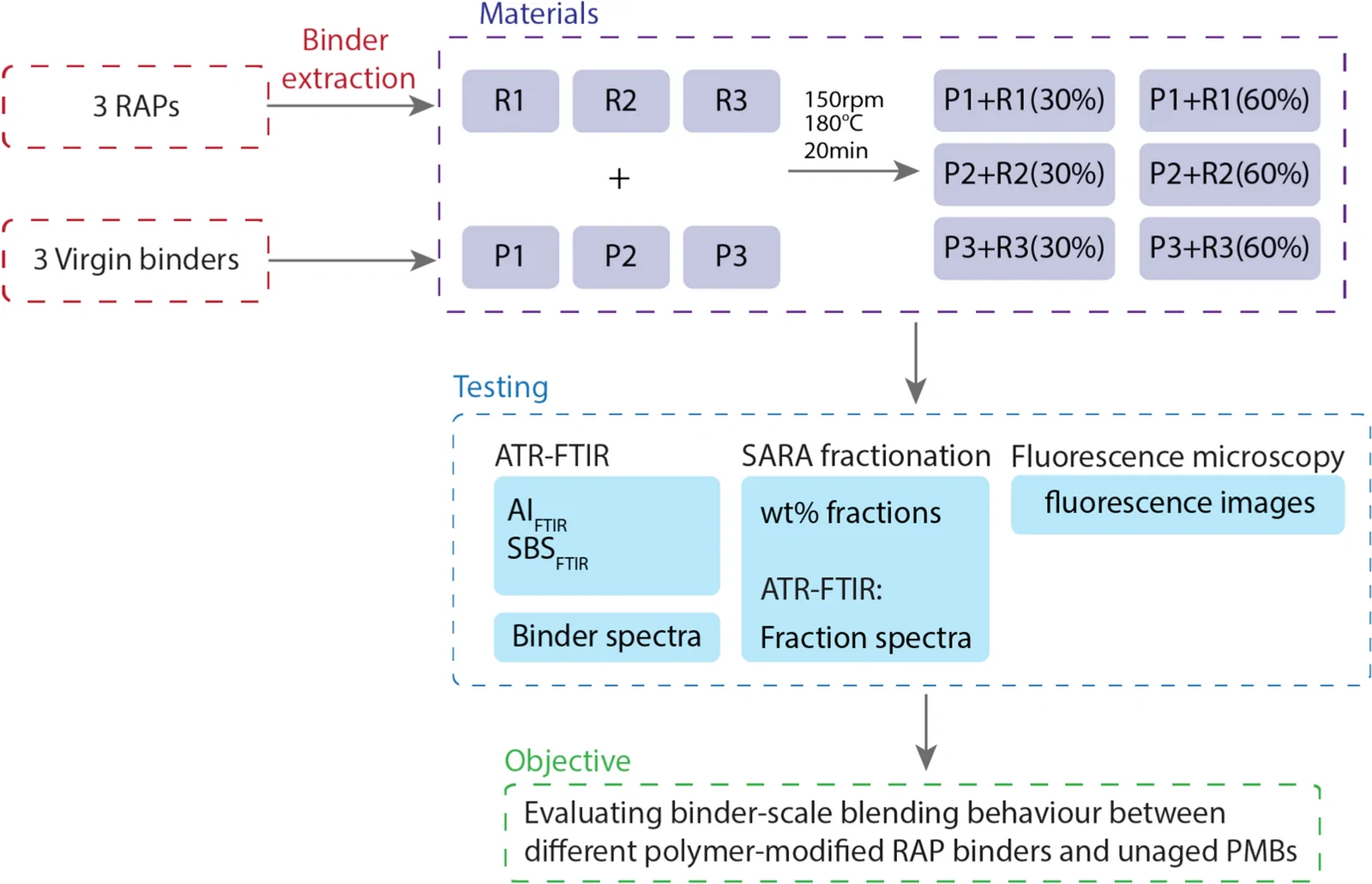
Flowchart of experimental methods

The objective of this study is to understand the influence of different PM-RAP contents on the chemical and morphological blending behaviour between PM-RAP and fresh PMB. To reflect real-world variability in both RAP and PMB sources, three RAP sources were selected from field sections in Austria, Belgium, and Switzerland originating from either stone mastic asphalt (SMA) or porous asphalt (PA) mixtures. From each location, PM-RAP binders were extracted and paired with a corresponding commercial PMB, representative of that country. Blends were prepared with 30% and 60% PM-RAP binder content. To evaluate the extent of blending and how it is affected by the PM-RAP content, a chemo-morphological characterisation was conducted on all individual binders and their respective blends. This included SARA fractionation, FTIR spectroscopy and fluorescence microscopy. The research approach is summarised in Fig. 1.

## Materials and methods

### Binder sources

In this study, three commercial SBS-modified PMBs were used in combination with three different PM-RAP binders. For simplicity, PMB is abbreviated as ‘P’ and PM-RAP binder as ‘R’. Each PM-RAP binder was extracted using tetrachloroethylene according to EN 12697-1 [29] and EN 12697-3 [30]. The specifications of all materials are given in Table 1.

**Table 1:** Summary of binder specifications

Name	Grading	Penetration (0.1 mm)	Softening point (°C)	Geographical origin	Mixture type
P1	45/80-65	50	77.9	Austria	–
P2	45/80-50	66	53.4	Belgium	–
P3	45/80-65	65	84.8	Switzerland	–
R1	–	9	76.0	Austria	SMA
R2	–	10	76.9	Belgium	SMA
R3	–	17	68.3	Switzerland	PA

### Binder blending

Each PM-RAP binder was blended with a commonly used commercial PMB from the same region, using two mixing ratios: 70/30 and 40/60 (PMB to PM-RAP binder, respectively). The constituent binders were first pre-heated to 180 °C. After reaching the target temperature, the appropriate amounts were weighed and combined. Blending was performed using an anchor-type impeller at 150 rpm for 20 min, while maintaining the temperature at 180 °C to ensure homogeneity. It should be noted that this blending procedure represents full binder blending under controlled laboratory conditions. In asphalt mixtures, however, full mobilisation of the RAP binder is not always achieved, and a fraction of the binder may remain inactive.

An overview of all samples is given in Table 2.

**Table 2:** Summary of blend compositions

Name	PMB (%)	RAP (%)
P1	100	0
P1+R1(30%)	70	30
P1+R1(60%)	40	60
R1	0	100
P2	100	0
P2+R2(30%)	70	30
P2+R2(60%)	40	60
R2	0	100
P3	100	0
P3+R3(30%)	70	30
P3+R3(60%)	40	60
R3	0	100

### SARA fractionation

All samples were separated into their four SARA fractions using the solid phase extraction principle reported previously [31]. In this method, a bitumen sample of 400 ± 20 mg was first dissolved in 40 ml of *n*-heptane and stirred for 24 ± 2 h. Then, the asphaltenes were extracted by using a 0.2 µm pore size filter (Thermo Scientific™ Titan3™ PTFE) attached to a vacuum manifold. The maltenes were separated into three fractions by injecting 15 ml of maltene solution with a concentration of 3.33 mg/mL onto a solid phase extraction (SPE) silica cartridge (Thermo Scientific™ HyperSep™, 40–60 µm particle size, 25 mL volume), which was first pre-washed. The fractions were then separated using a series of solvents, as listed in Table 3. The solvents of each resulting fraction were evaporated by a combination of a heating plate and continuous nitrogen (N_2_) flow. Finally, the weight percentage (wt%) of each fraction was determined gravimetrically. The polarity index was calculated according to Eq. (1), where higher values correspond to a higher proportion of polar fractions [24, 32]. The Gaestel index was calculated according to Eq. (2), where higher values correspond to increased colloidal instability [28].

**Table 3:** Overview of the used solvents for maltene separation and their ratios

Fraction	Solvent	Ratio (%)	Quantity (mL)
Prewash	*n*-heptane	100	20
Saturates	*n*-heptane	100	10
Aromatics	Toluene:*n*-heptane	80:20	25
Resins	Dichloromethane:Methanol	90:10	40

1 $$\mathrm{P}\mathrm{o}\mathrm{l}\mathrm{a}\mathrm{r}\mathrm{i}\mathrm{t}\mathrm{y}\text{ } \mathrm{i}\mathrm{n}\mathrm{d}\mathrm{e}\mathrm{x}=\frac{(\mathrm{R}\mathrm{e}\mathrm{s}\mathrm{i}\mathrm{n}\mathrm{s}+\mathrm{A}\mathrm{s}\mathrm{p}\mathrm{h}\mathrm{a}\mathrm{l}\mathrm{t}\mathrm{e}\mathrm{n}\mathrm{e}\mathrm{s})}{(\mathrm{S}\mathrm{a}\mathrm{t}\mathrm{u}\mathrm{r}\mathrm{a}\mathrm{t}\mathrm{e}\mathrm{s}+\mathrm{A}\mathrm{r}\mathrm{o}\mathrm{m}\mathrm{a}\mathrm{t}\mathrm{i}\mathrm{c}\mathrm{s})}$$

2 $$\mathrm{G}\mathrm{a}\mathrm{e}\mathrm{s}\mathrm{t}\mathrm{e}\mathrm{l}\text{ } \mathrm{i}\mathrm{n}\mathrm{d}\mathrm{e}\mathrm{x}=\frac{(\mathrm{A}\mathrm{s}\mathrm{p}\mathrm{h}\mathrm{a}\mathrm{l}\mathrm{t}\mathrm{e}\mathrm{n}\mathrm{e}\mathrm{s}+\mathrm{S}\mathrm{a}\mathrm{t}\mathrm{u}\mathrm{r}\mathrm{a}\mathrm{t}\mathrm{e}\mathrm{s})}{(\mathrm{R}\mathrm{e}\mathrm{s}\mathrm{i}\mathrm{n}\mathrm{s}+\mathrm{A}\mathrm{r}\mathrm{o}\mathrm{m}\mathrm{a}\mathrm{t}\mathrm{i}\mathrm{c}\mathrm{s})}$$

### Fourier-transform infrared (FTIR) spectroscopy

ATR-FTIR was used to assess changes in functional groups upon blending. A Bruker Alpha II FTIR spectrometer recorded spectra in the range of 4000–400 cm^−1^. Four samples per binder were measured with a resolution of 4 cm^−1^ and 24 scans. Each sample was scanned four times, resulting in a total of 16 spectra per binder. All spectra were processed using the OPUS software. A min–max normalisation was applied based on the aliphatic hydrocarbon band at 2920 cm^−1^, followed by the integration of functional groups. The change in bitumen oxidation state upon blending was evaluated using an ageing index shown in Eq. (3) [24], while the polymer was tracked via characteristic SBS functional groups displayed in Eq. (4) [11].

- *Carbonyls (A*_*CO*_*)* full baseline integration 1800–1660 cm^−1^.
- *Sulfoxides (A*_*SO*_*)* full baseline integration 1079–984 cm^−1^.
- *Polarity shift (A*_*PO*_*)* full baseline integration 1330–1130 cm^−1^.
- *Polybutadiene 1,4-trans-alkene (A*_*PB-trans*_*)* valley-to-valley integration 979–950 cm^−1^.
- *Polybutadiene 1,2-vinyl (A*_*PB-vinyl*_*)* valley-to-valley integration: 915–903 cm^−1^.
- *Polystyrene monoalkylated aromatic (A*_*PS*_*)* valley-to-valley integration 707–690 cm^−1^.
  3 $${\mathrm{A}\mathrm{I}}_{\mathrm{F}\mathrm{T}\mathrm{I}\mathrm{R}}={A}_{\mathrm{C}\mathrm{O}}+{A}_{\mathrm{S}\mathrm{O}}+{A}_{\mathrm{P}\mathrm{O}}$$
  4 $${\mathrm{S}\mathrm{B}\mathrm{S}}_{\mathrm{F}\mathrm{T}\mathrm{I}\mathrm{R}}={A}_{\mathrm{P}\mathrm{B}-\mathrm{t}\mathrm{r}\mathrm{a}\mathrm{n}\mathrm{s}}+{A}_{\mathrm{P}\mathrm{B}-\mathrm{v}\mathrm{i}\mathrm{n}\mathrm{y}\mathrm{l}}+ {A}_{\mathrm{P}\mathrm{S}}$$

SARA fractions were analysed by smearing them directly onto the crystal, while asphaltenes were first dissolved in dichloromethane, then dropped onto the crystal and measured after evaporation of the solvent. All spectra were normalised using the same procedure as applied to the binders.

### Fluorescence microscopy

A Nikon Optical Inverse Microscope (OIM) was used in fluorescence mode to observe the polymer phase. The microscope is equipped with a Märzhäuser *X*-*Y*-*Z* stage, Nikon DS-Fi3 camera, an epifluorescence unit and a Nikon CFI60 TU Plan Epi ELWD 50X (NA/WD: 0.60/11.0 mm). A 435 nm LED from the CoolLED pE-4000 was used for illumination (the lamp contains 15 LEDs in the wavelength range: 365–770 nm). The setup included a custom fluorescence filter block with a 403/95 nm excitation filter (353–452 nm), a 495 nm dichroic mirror, and a 500 nm long-pass emission filter. Samples were prepared by applying a hot binder drop on a heated microscopy slide at 150 °C. A cover slide was placed on the drop and left for around 1 min to create a thin layer of bitumen. Images were captured at this initial state to observe the polymer dispersion. To better visualise the polymer phase, phase separation was induced by heating the slide to 120 °C at a heating rate of 50 °C/min and leaving it isothermally for 1 h using a Linkam LTS420 heating stage [26]. Images were captured using NIS-Elements AR software with an exposure time of 900 ms. To compensate for surface unevenness, a z-stack (± 10 µm around the focal plane) was taken and merged into a fully focused composite image.

## Results and discussion

### Fourier-transform infrared (FTIR) spectroscopy

**Fig. 2:**
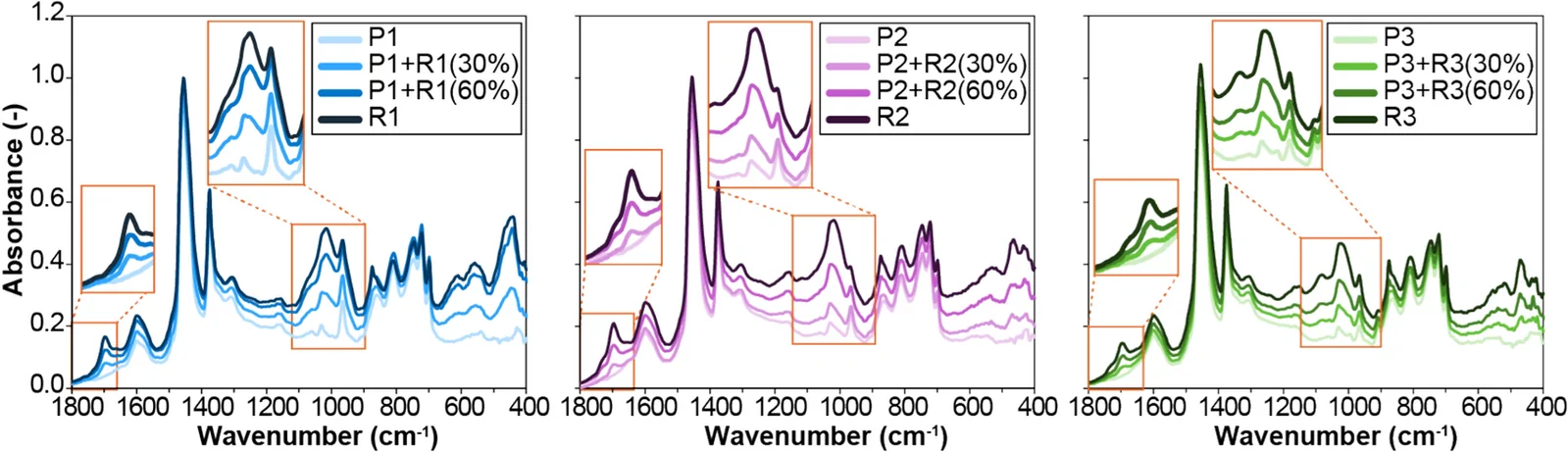
FTIR spectra of all binder blends in the extended fingerprint region (400–1800 cm^−1^) where a darker colour indicates a higher percentage of PM-RAP binder

**Fig. 3:**
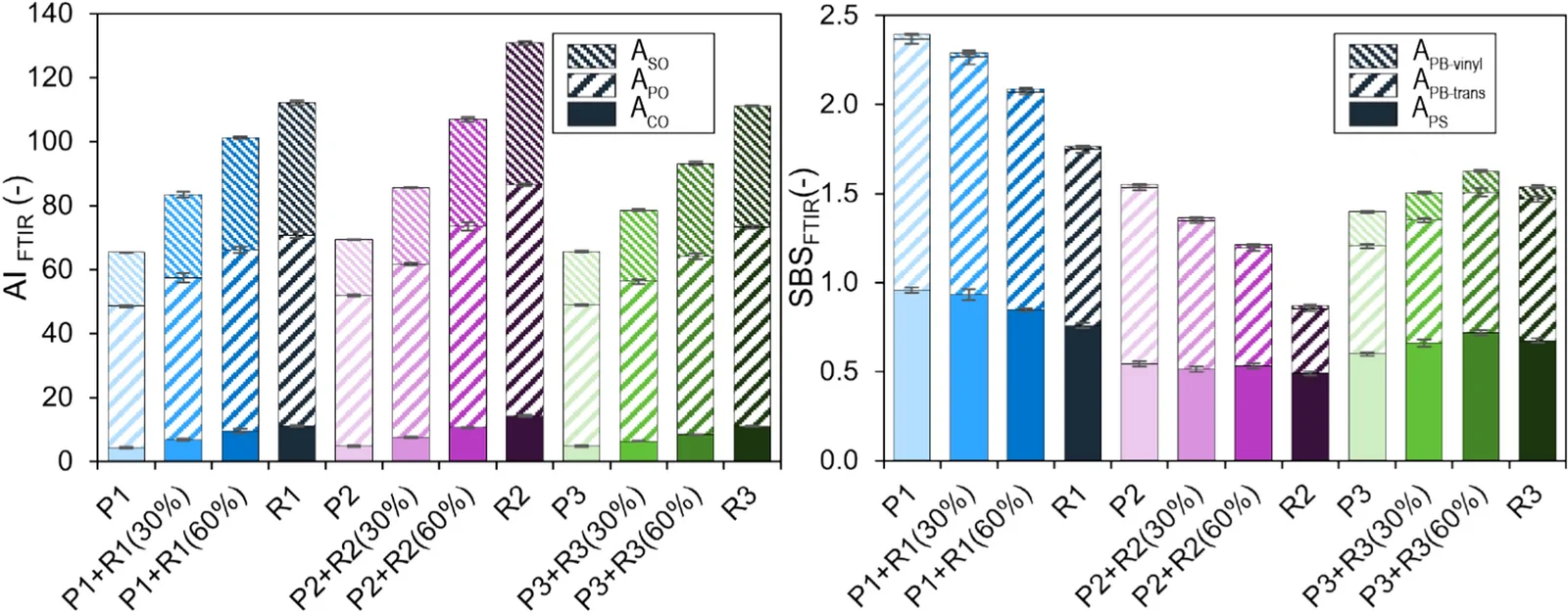
FTIR binder ageing indexes (left) and polymer indexes (right) for all blends

Figure 2 shows the extended fingerprint region of all blends to visualise chemical changes induced by the addition of PM-RAP binder. Figure 3 shows the semi-quantitative determination of bitumen and polymer indexes.

In general, the addition of PM-RAP binder altered the spectra depending on the source and ageing level of the PM-RAP binder. Specifically, blends containing R2 and R3 exhibited a shoulder around 1735 cm^−1^ corresponding to carboxylic acid groups typically associated with UV-induced oxidation [33, 34]. This highlights the need to consider UV-related or in general sunlight-induced ageing in surface layers when recycling field-aged binder, as standard laboratory ageing does not capture these effects. The carbonyl (~ 1700 cm^−1^), aromatic (~ 1600 cm^−1^) and sulfoxide (~ 1030 cm^−1^) regions showed increased absorbance upon PM-RAP addition, comparable to oxidative ageing. This was also reflected by the increase in the AI_FTIR_ index, confirming a higher overall oxidation level in the blends compared to the virgin binders.

Signals around 699 and 966 cm^−1^, corresponding to polystyrene and polybutadiene C–H bending in SBS polymers, were present in all virgin PMBs but changed in intensity upon PM-RAP addition as indicated by SBS_FTIR_. In blends with R1 and R2, these bands decreased in absorbance, reflecting the diluting effect of adding more aged or polymer-degraded PM-RAP binders. In contrast, the polymer bands in the P3+R3 blends increased slightly upon addition of the PM-RAP binder, as R3 exhibits a higher SBS_FTIR_ signal compared to P3. Additionally, P3 exhibited a generally lower intensity band in the polybutadiene-associated region (966 cm^−1^) than P1 and P2, and a more distinct vinyl group band at 910 cm^−1^. Important to note is the presence of remaining filler particles, which can be identified by the 874 cm^−1^ band. Their impact on the overall spectrum becomes clearer in the asphaltene analysis, where all characteristic bands are presented.

Despite these changes, no new absorption bands were observed after blending, indicating that the blending process results in a physical mixture of chemical components rather than the formation of new compounds. Although FTIR provides valuable information on chemical changes, it cannot fully describe the condition of the SBS network, as it only reflects semi-quantitative variations in functional groups.

### Quantitative analysis of SARA fractions

Blending new PMB with PM-RAP binder creates a complex system, made up of both aged and unaged base binders, as well as aged and unaged polymers. Several studies [35–38] suggest that the bitumen composition, especially the SARA fractions (saturates, aromatics, resins, and asphaltenes), plays an important role in polymer-bitumen compatibility. In particular, the lighter components like saturates and aromatics are thought to be key for ensuring proper interaction with the polymer and for maintaining good mechanical properties [2].

During ageing, the aromatic components in bitumen will decrease, moving towards resins and subsequently evolving to asphaltenes [24, 39]. This loss of light components in the reclaimed binder can shift the overall SARA balance and alter the colloidal structure of the bitumen. When PM-RAP binder is added to virgin PMB, this change in composition may disrupt the original balance of SARA fractions, reduce the effectiveness of polymer dispersion, and compromise the performance of the modified binder. As a result, evaluating the SARA fractions can provide valuable insight into the chemical compatibility and stability of blends containing reclaimed PMB.

**Fig. 4:**
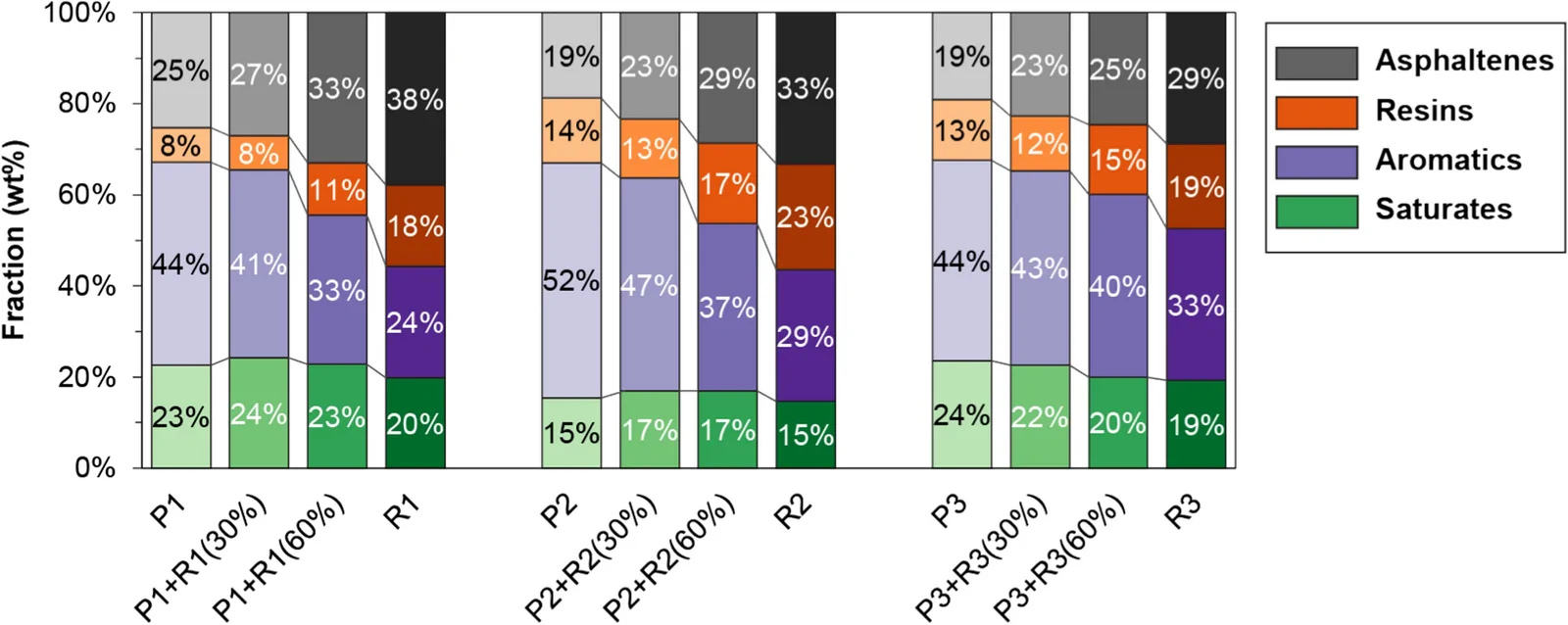
Quantified SARA Fractions for each blend

The wt% of the SARA fractions for each binder and blend is shown in Fig. 4. With increasing PM-RAP binder content, the weight percentage of asphaltenes and resins increased, while the weight percentage of aromatics and saturates decreased. This means a shift to higher polarity, similar to what happens during the ageing of bitumen [24], as reflected by the increasing polarity index in Table 4, due to the addition of already oxidised molecules. The increasing Gaestel index with increasing RAP content indicates a trend from a more sol-type system toward a more gel-type system, which is thought to be less stable. To better understand chemical changes during the blending of PMB with PM-RAP, FTIR was performed on each fraction.

**Table 4:** Polarity and Gaestel indices of all blends

Binder	Polarity index	Gaestel index
P1	0.65	0.92
P1+R1(30%)	0.69	1.05
P1+R1(60%)	0.91	1.26
R1	1.31	1.36
P2	0.62	0.52
P2+R2(30%)	0.68	0.68
P2+R2(60%)	0.93	0.84
R2	1.28	0.92
P3	0.73	0.74
P3+R3(30%)	0.73	0.83
P3+R3(60%)	0.83	0.80
R3	1.04	0.92

### FTIR analysis of SARA fractions

FTIR analysis of the individual SARA fractions provides insight into the chemical composition of the component binders and reveals changes that occur during blending. It allows for a more detailed comparison between different RAP and PMB sources and helps to better understand the transformations that take place when they are mixed. In addition, it clarifies which fraction the polymer is found in during binder fractionation using liquid chromatography methods, which until now has remained largely unexplored.

#### Asphaltenes

**Fig. 5:**
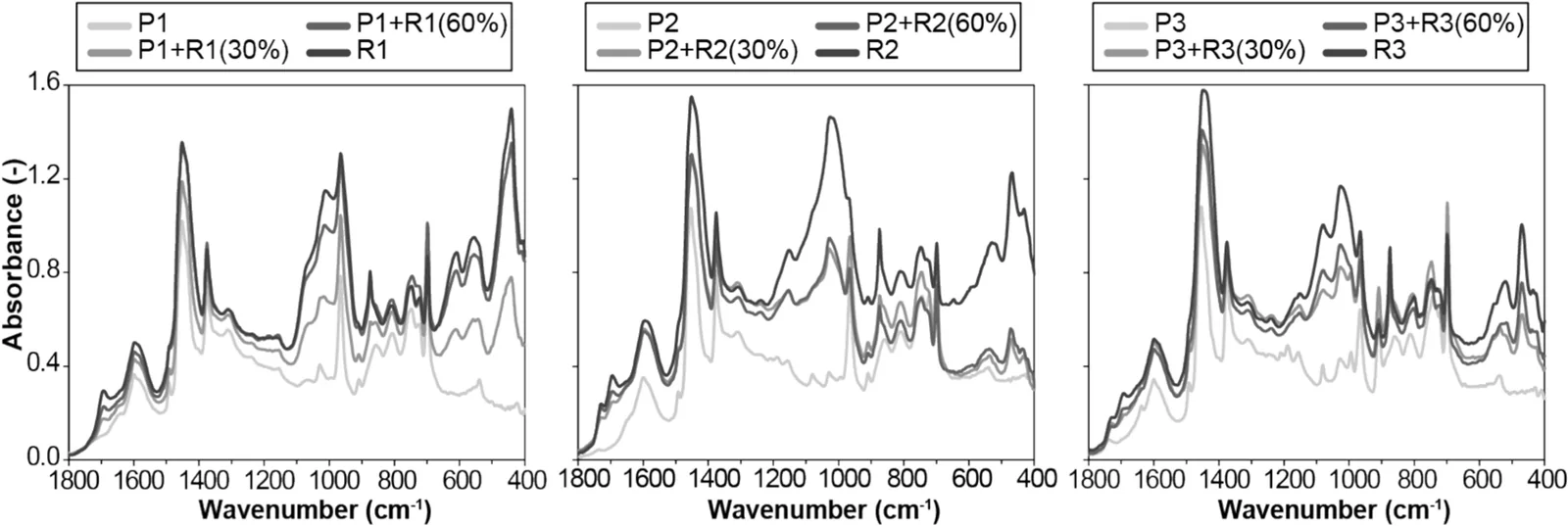
FTIR spectra of the asphaltene fraction of all blends

The FTIR spectra of the fingerprint region of the asphaltenes are shown in Fig. 5. An overview of all major functional groups can be found elsewhere [24]. Starting from high wavenumbers, a clear increase in carbonyl functional groups was observed upon adding PM-RAP binder. The signal at 1735 cm^−1^ is present here as previously seen in the bitumen spectra and corresponds to carboxylic acids (esters). Only blends containing R1 did not exhibit this signal.

The region between 1350 and 1000 cm^−1^, belonging to multiple sulphur-containing functional groups, also showed a steep increase upon adding PM-RAP binder. However, this region can also be influenced by residual mineral filler particles. In particular, signals at 874 cm^−1^, which are attributed to calcite, confirm the presence of limestone filler. Calcite also exhibits characteristic bands at 1420 and 712 cm^−1^. Limestone is rarely composed of pure calcite, it typically includes other mineral phases such as quartz, dolomite (1446, 881, 730 cm^−1^), and clays (3689, 3627, 1033, 1010, 912 cm⁻^1^) [40].

The polymer was primarily found in the n-heptane insoluble phase, which aligns with SBS’s partial solubility in *n*-heptane documented in literature [41]. The main SBS-related absorptions at 966 cm^−1^ (trans-butadiene), 910 cm^−1^ (vinyl double bond) and 699 cm^−1^ (styrene ring deformation) were present with high absorbance. Compared to the spectra of the virgin binders, the vinyl signal at 910 cm^−1^ appeared more distinct, indicating that fractionation provides deeper insights into the polymer structure. This band was present in all binders but generally showed the lowest absorption in the PM-RAP binders. Its intensity decreased further with increasing PM-RAP binder content. A high concentration of vinyl double bonds has been known to enhance thermal stability, as scission at the double bond does not break the polymer backbone. Moreover, polymer–bitumen interactions can be improved due to reduced steric hindrance associated with the C=C double bond [42, 43]. Thus, this ‘dilution’ could affect the thermal stability of the resulting blends and reduce their resistance to further ageing. This means that blends with P3 and R3 might have superior performance.

It must be noted that baseline differences were likely due to remaining filler in the asphaltene phase caught by the filter and/or the solid-state nature of the asphaltenes, which may lead to scattering due to poor contact with the crystal, changes in refractive index, or Mie scattering [44, 45].

#### Resins

**Fig. 6:**
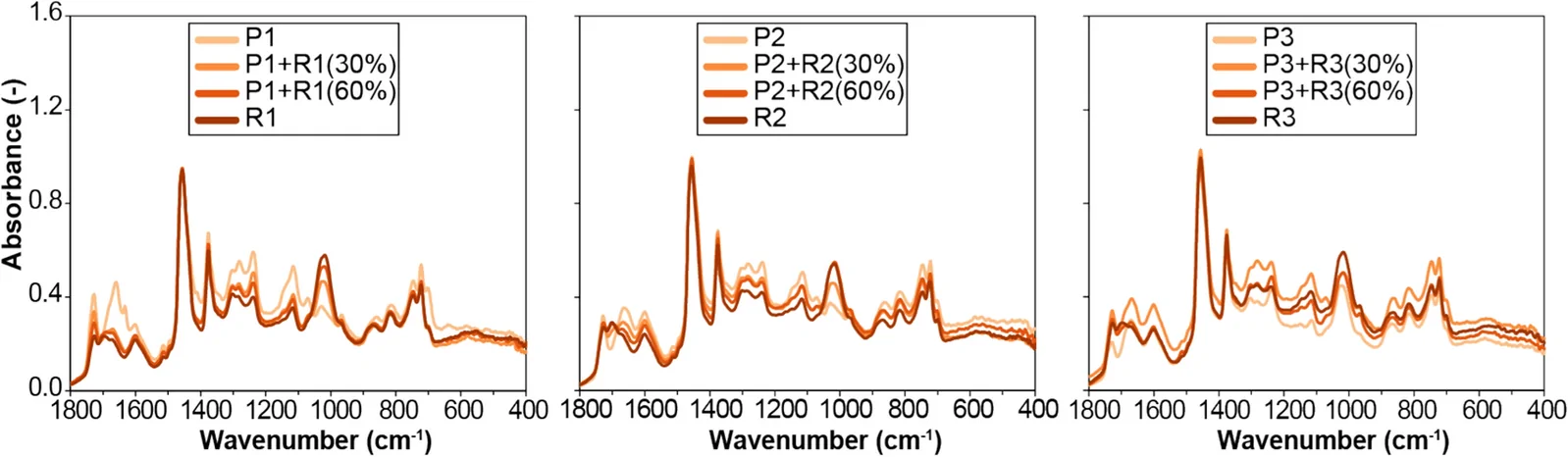
FTIR spectra of the resin fraction of all blends

The FTIR spectra of the extended fingerprint region of the resins are shown in Fig. 6. Starting from the carbonyl area between 1800 and 1660 cm^−1^. With addition of PM-RAP binder the bands at 1735 cm^−1^ (carboxylic acids) and 1655 cm^−1^ (2-quinolones) decreased, and the band at 1700 cm^−1^ (ketone) slightly increased. It is also worth mentioning that P1 seemed to have a higher quinolone concentration compared to the other PMBs, most probably due to differences in crude oil source. Similar to asphaltenes, the sulfoxides at 1030 cm^−1^ also increased with higher PM-RAP binder percentage. Low-intensity SBS bands (966, 699 cm^−1^) were identified. This suggests that either part of the polymer is dissolved in the maltenes, or that some polymer molecules are small enough to pass through the filter used to separate the asphaltenes.

#### Aromatics

**Fig. 7:**
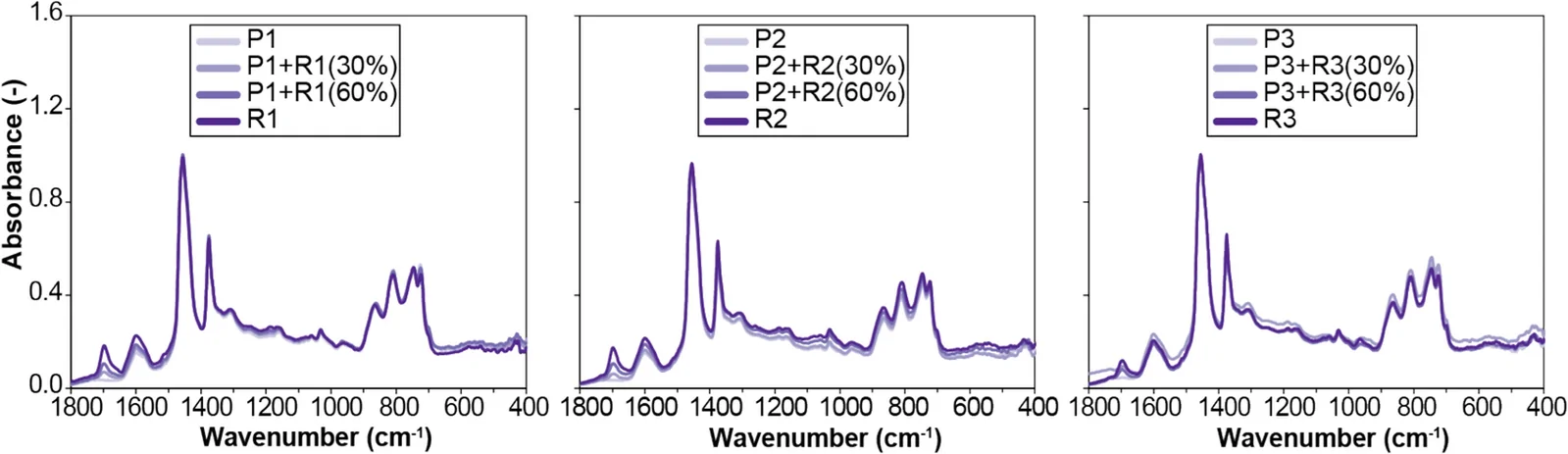
FTIR spectra of the aromatic fraction of all blends

The FTIR spectra of the fingerprint region of the aromatics are shown in Fig. 7. Upon addition of PM-RAP binder, there was a clear increase of the 1700 cm^−1^ (ketones) band. For blends including R1 and R2, the aromatic band at 1600 cm^−1^ increased for higher PM-RAP binder percentages which is a sign of ageing in the PM-RAP binder or alternatively differences in crude oil origin. Also here, low-intensity SBS bands (966, 699 cm^−1^) were present.

#### Saturates

**Fig. 8:**
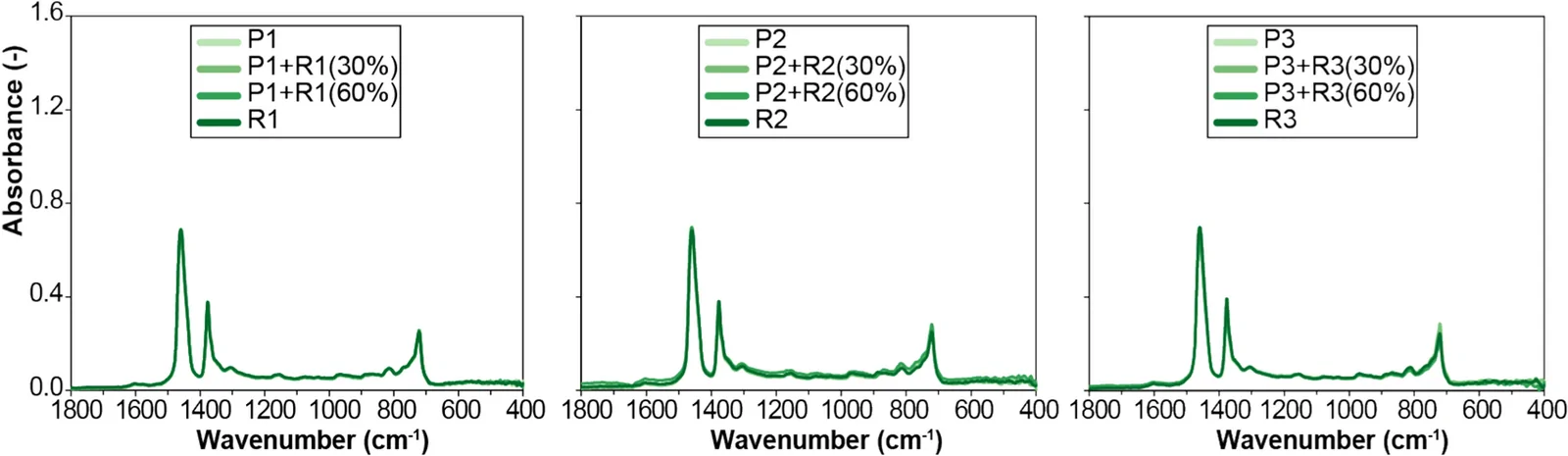
FTIR spectra of the saturate fraction of all blends

Saturates consist mainly of non-polar, low molecular weight, aliphatic hydrocarbons. They are known to be chemically inert [28]. This was confirmed by the limited change in the spectra upon PM-RAP binder addition, as illustrated by Fig. 8. No clear SBS signal was present. Even though SBS is partially soluble in *n*-heptane, the polymer that passes through the filter during the asphaltene separation step doesn’t seem to end up in the saturates fraction.

### Experimental versus theoretical ratios and practical implications

**Fig. 9:**
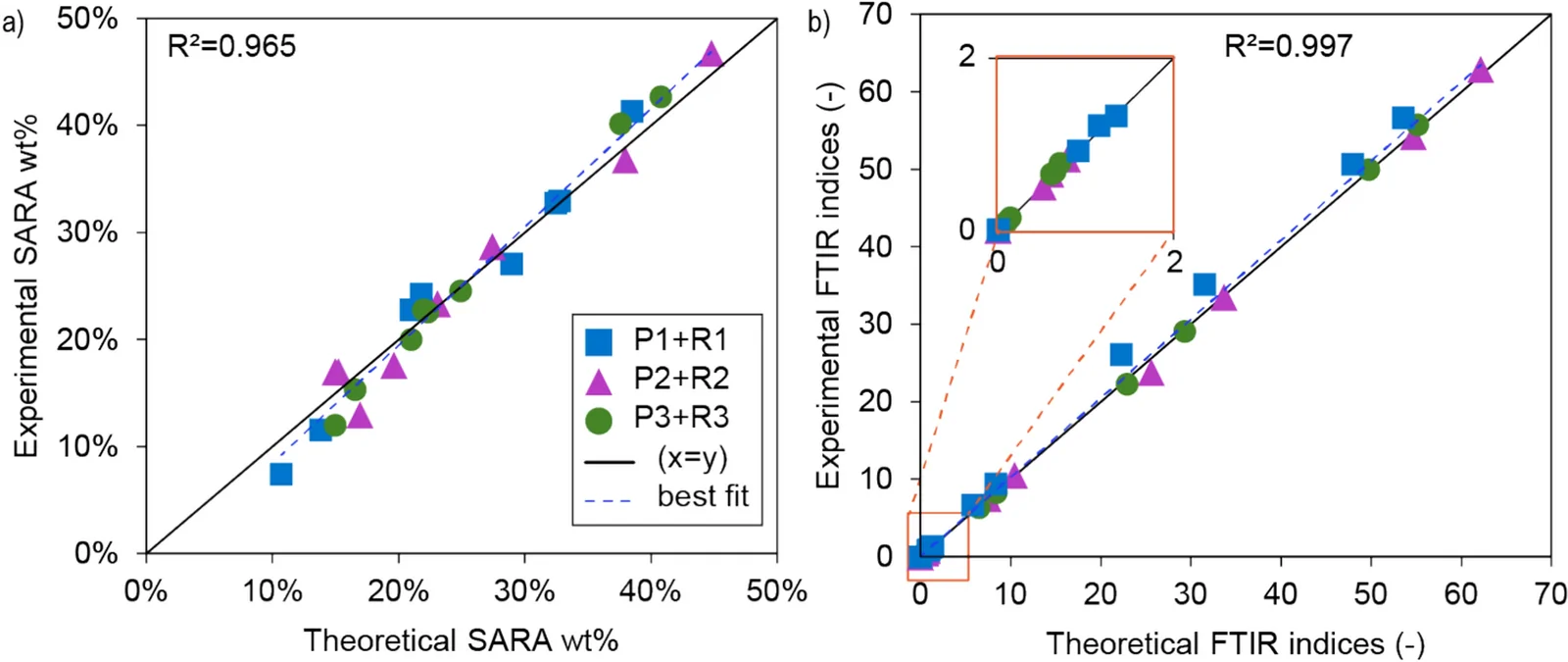
Experimental vs. theoretical values of **a)** SARA wt% and **b)** FTIR indexes

Figure 9 shows scatter plots of the experimental values of the SARA wt% and FTIR indexes versus the theoretically calculated values of the blends. These theoretical values were derived from the measured properties of the individual constituent binders: virgin PMBs (P1, P2, P3) and PM-RAP binders (R1, R2, R3), according to their blend ratios (e.g., 70% P1 and 30% R1). It should be noted that these calculations assume full blending of the constituent binders. In asphalt mixtures, however, full mobilisation of the RAP binder is not always achieved. Nevertheless, if the effective degree of blending can be estimated, this approach could still be applied by adjusting the blend ratios to reflect the proportion of RAP binder that actively participates in the blended binder.

Comparison of calculated versus experimental SARA fractions and FTIR indexes shows good agreement with the theoretical *X* = *Y* line, with *R*^2^ = 0.965 for SARA and *R*^2^ = 0.997 for FTIR. This suggests that the blend properties can be reliably estimated based on the properties of the input materials. This is particularly valuable in the context of recycling, as it enables a predictive approach to blend design. If the SARA fractions and FTIR indexes can be estimated in advance, the composition can be adjusted accordingly by adding components such as solvating oils or aromatic fractions to achieve the desired chemical balance and binder performance.

However, while the chemical composition can be estimated, the formation of the polymer network in the final binder remains difficult to predict. These aspects are critical for the mechanical performance of PMB blends and can be investigated through morphological analysis. Therefore, fluorescence microscopy was used to visualise the polymer phase in virgin, aged, and blended binders.

### Fluorescence microscopy

**Fig. 10:**
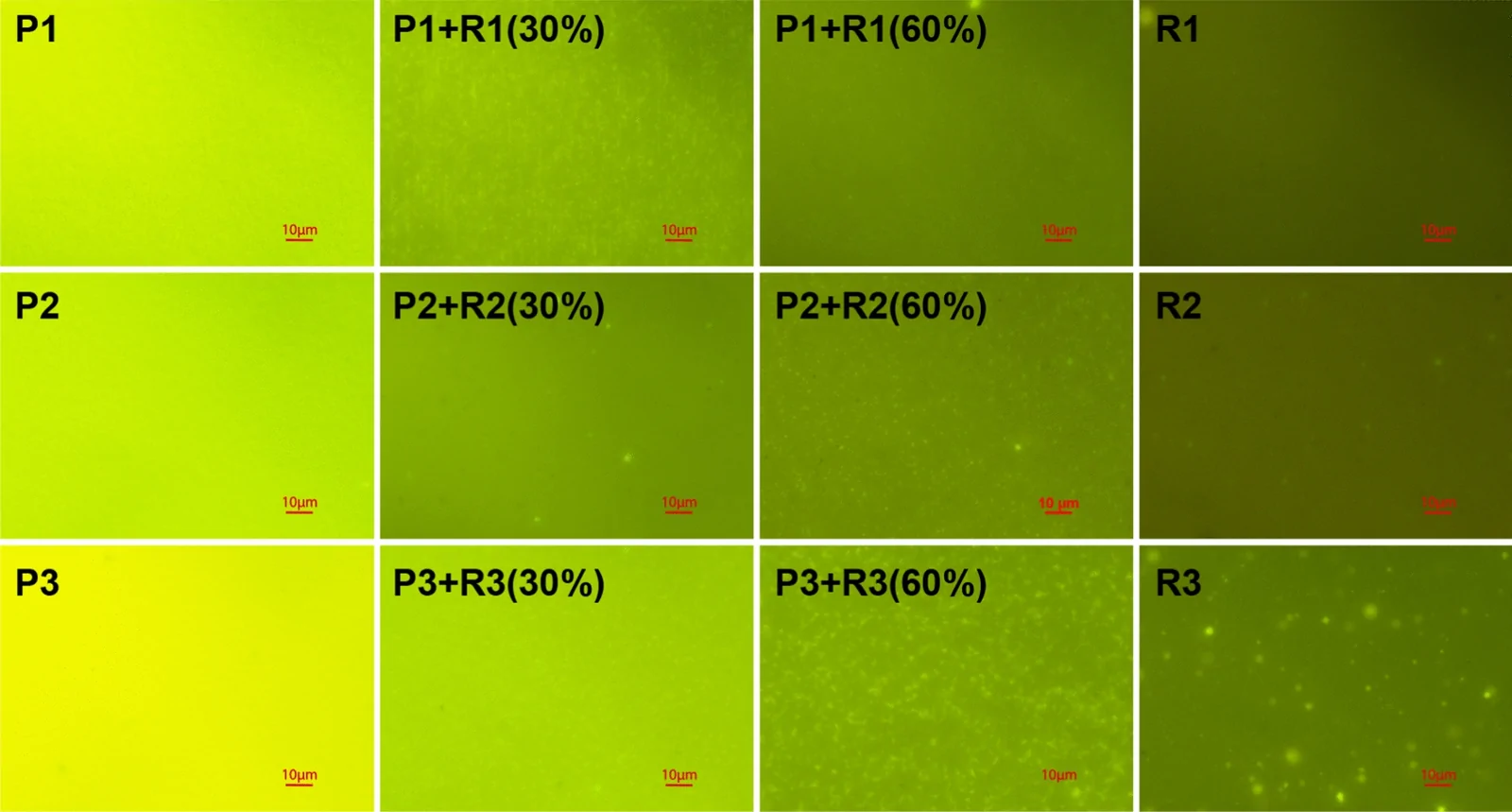
Fluorescence micrographs (50 × magnification) of all binder samples before phase separation, prepared with a cover slide

Figure 10 shows all binders and their blends’ micrographs visualised after blending but before inducing phase separation. Generally, homogeneous blends could be achieved using the method described in this work. The virgin PMBs (P1, P2 and P3) showed strong fluorescence with the absence of a clearly distinguishable polymer phase, indicating good polymer miscibility. The PM-RAP binders (R1, R2, R3), in contrast, exhibited significantly weaker fluorescence and a more heterogeneous morphology. Notably, R3 displayed bright spots, suggesting that the polymer was not degraded to the extent that it is fully dissolved into the bitumen matrix. This interpretation is supported by previous studies, which show that ageing of PMBs typically leads to a transition from a well-dispersed polymer network to smaller, isolated domains, whereas more severe ageing can result in uniform dispersion or complete polymer depletion [46–48].

In the blends with R2 and R3 (second and third row Fig. 10), increasing RAP content from 30 to 60% resulted in more visible, isolated fluorescent domains, suggesting worsening miscibility and compatibility of the polymer-bitumen system. For blends with R1 (top row Fig. 10) the opposite was observed. Due to the decent miscibility of the polymers in these blends, a phase separation step was necessary to better visualise and distinguish the polymer phase.

**Fig. 11:**
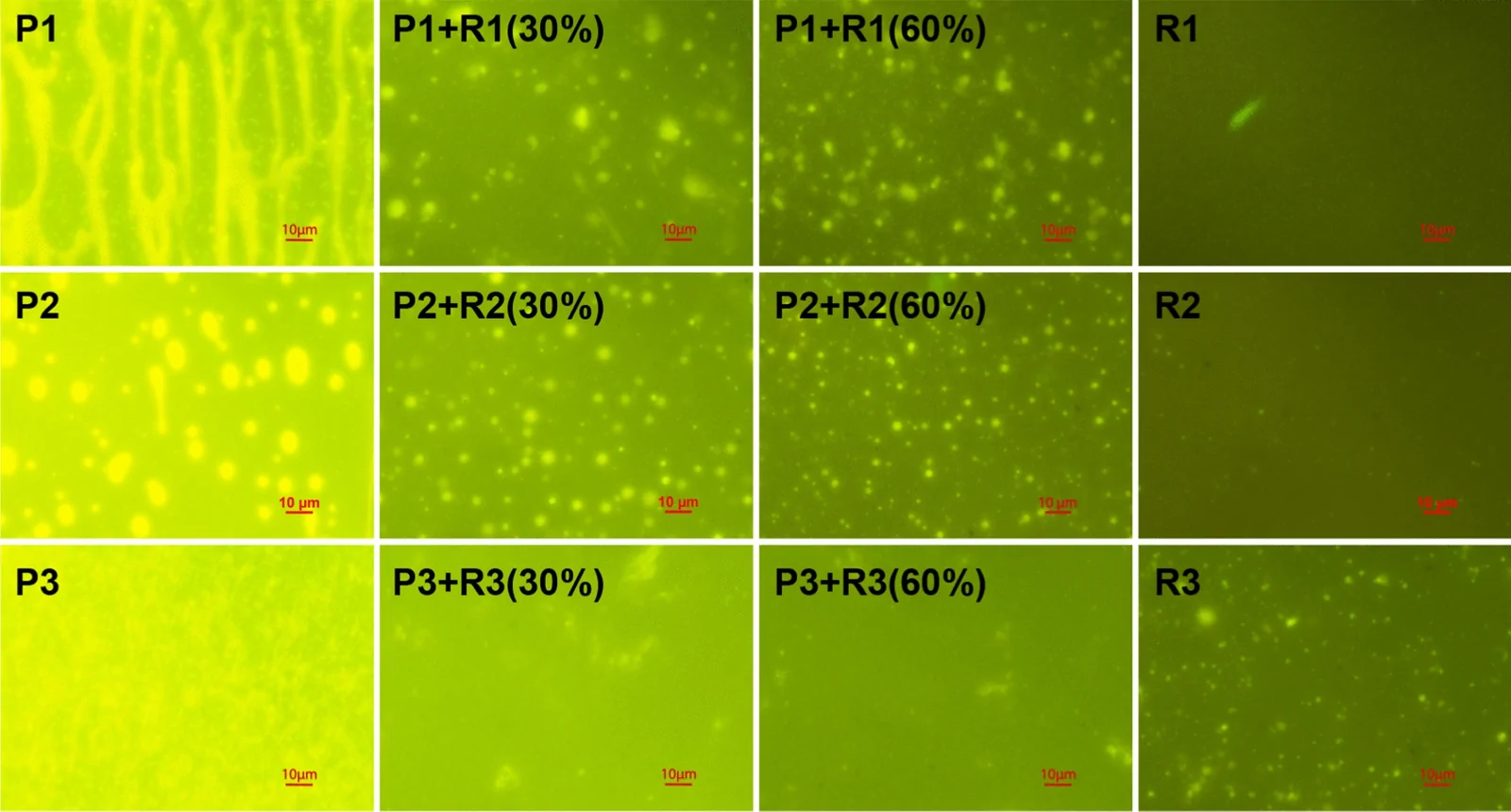
Fluorescence micrographs (50 × magnification) of all binder samples after isothermal phase separation at 120 °C for 1 h

The phase-separated binders are represented by Fig. 11. In these images the polymer phase became more clear and differences were easier to distinguish. Clear differences were observed among the virgin binders (P1, P2, and P3). P1 and P2 had distinct polymer domains, while P3 had a more finely dispersed polymer network, even after thermal phase separation. This suggests that the polymer in P3 had good compatibility with the base binder. Since P3 also exhibited a higher vinyl area in the FTIR characterization, this is hypothesized to contribute to the improved compatibility. For the PM-RAP binders, R1 and R2 still displayed homogeneous morphologies, indicating significant degradation of the polymer phase. R3 on the other hand, showed even more polymer domains after phase separation, suggesting a lower ageing degree and the potential for the remaining polymer to still contribute to the mechanical performance of the binder.

For the blends, two general trends could be seen with increasing PM-RAP percentages: 1) a reduction in overall fluorescence intensity 2) a decrease in the size of the polymer domains. These trends could be attributed to the incorporation of aged binder with diminished fluorescence response (explaining the lower intensity) and dilution of the virgin polymer (explaining the smaller domain sizes), particularly in cases where the PM-RAP binders (e.g., R1 and R2) contain highly degraded polymer. The system containing P3 and R3 is more complex. Here, even after 1 h at 120 °C, the polymer phase was only slightly visible in cloud-like domains. These observations suggest that, although the binders appear homogeneous prior to phase separation, increasing PM-RAP content reduces the relative contribution of the virgin polymer to the overall binder structure. The reduction in fluorescence can be linked to a reduction of the virgin polymers and an increase of the aged polymers. This implies the presence of smaller polymer particles [14] and a more diluted polymer network in the final blend.

## Summary and conclusions

This study evaluated the chemical and morphological blending phenomena between polymer-modified RAP binder and unaged SBS-modified binder at 30% and 60% PM-RAP binder replacement levels. The key findings of this research are summarised as follows:

- ATR-FTIR analysis showed that the presence of PM-RAP binder in the analysed blends increases the ageing index (AI_FTIR_) due to the higher oxidation level of the PM-RAP. SBS-related signals vary depending on the PM-RAP source, either increasing or decreasing in absorbance. Nevertheless, FTIR showed limitations for assessing the polymer network, as it only reflects changes in functional groups rather than the point at which the SBS network becomes fully disrupted.
- SARA fractionation revealed a shift in chemical composition with increasing RAP content: saturates and aromatics decrease, while resins and asphaltenes increase, similar to the ageing of bitumen.
- FTIR analysis of the SARA fractions showed that SBS primarily concentrates in the *n*-heptane insoluble phase together with the asphaltenes, with traces in the resins and aromatics. The addition of PM-RAP binder alters chemical functionalities across all fractions, particularly increasing signals in the carbonyl and sulfoxide-related regions, while the saturates remain largely unaffected.
- Chemical analyses suggest that blending occurs primarily through physical mixing of chemical components.
- The strong correlation observed between experimental and calculated blend properties suggests that the chemical composition of binder blends can be reliably estimated from the input materials. This enables a predictive approach to recycling.
- Fluorescence microscopy showed an overall reduction in fluorescence intensity and a decrease in polymer domain size upon PM-RAP binder addition. Morphology varied across binders and blends, reflecting differences in PMB formulations and PM-RAP sources.

These findings provide new insights into the chemical and morphological blending mechanisms between aged polymer-modified RAP binders and unaged SBS-modified binders. The results highlight that blending is primarily physical and strongly influenced by the ageing state and origin of the PM-RAP binder. The strong correlation between experimental and calculated chemical blend properties is crucial for mix design. It means that blends could be designed without having to test each one in the lab, which is especially useful when planning to use additives. However, the noticeable differences in fluorescence morphology between binders and blends suggest that FTIR and SARA alone are not enough to describe blending. Future work should link these chemical and morphological observations to mechanical performance.

## Data Availability

The data of this study will be available upon request.
